# Effects of vitamin D supplementation on 25(OH)D concentrations and blood pressure in the elderly: a systematic review and meta-analysis

**DOI:** 10.12688/f1000research.24623.3

**Published:** 2020-09-03

**Authors:** Farapti Farapti, Chusnul Fadilla, Niwanda Yogiswara, Merryana Adriani

**Affiliations:** 1 Department of Nutrition, Faculty of Public Health, Universitas Airlangga, Surabaya, East Java, 60115, Indonesia; 2 Post Graduate Doctoral Program, Faculty of Medicine, Universitas Airlangga, Surabaya, Indonesia; 3 Faculty of Medicine, Universitas Airlangga, Surabaya, East Java, 60132, Indonesia

**Keywords:** vitamin D, blood pressure, elderly, 25(OH)D) levels

## Abstract

**Background**: Hypertension and vitamin D deficiency are prevalent among the elderly. This study evaluated the effects of vitamin D supplementation on changes in serum 25-hydroxyvitamin D (25(OH)D) concentrations and blood pressure (BP) in the elderly (age > 60 years).

**Methods**: Randomized controlled trials from electronic databases on the elderly taking oral vitamin D, until the end of March 2019, were selected. Two reviewers independently screened the literature on the basis of specific inclusion criteria. The primary outcomes were serum 25(OH)D level, systolic BP (SBP), and diastolic BP (DBP) changes.

**Results**: Our analysis revealed significant differences in serum 25(OH)D concentrations changes between the vitamin D and control groups (mean difference [MD] = 13.84; 95% confidence interval [CI] = 10.21–17.47; P < 0.000). There were no significant differences in SBP and DBP changes between the vitamin D and control groups. Subgroup analysis revealed significant differences in SBP changes between the hypertensive and vitamin D-deficient subgroups (MD = –4.01; 95% CI = –7.45 to –0.57; P = 0.02 and MD = –1.91; 95% CI = –3.48 to –0.34; P = 0.02, respectively), and DBP changes only in the hypertensive subgroup (MD = –2.22; 95% CI = –4.1 to –0.34; P = 0.02).

**Conclusions**: Vitamin D supplementation significantly increases 25(OH)D concentrations and seems beneficial in lowering BP, specifically in the elderly with elevated BP and vitamin D deficiency.

## Introduction

High blood pressure (BP), or hypertension, is still regarded as one of the most influential factors for cardiovascular diseases, especially in the elderly. An increasingly aging population and the increasing prevalence of hypertension emphasize the importance of proper treatment of hypertension. Nutrient supplementation is an alternative treatment since the elderly have multiple chronic diseases and take multiple drugs ^1, 2^. Vitamin D is one kind of steroid hormone and micronutrient synthesized in the skin by exposure to ultraviolet B rays and also obtained through dietary intake or supplementation ^3^. Most vitamin D is distributed in the human body in the form of serum 25(OH)D ^4^.

Vitamin D deficiency has become an important public health concern because it could take place at any age, and most countries report deficiency as high in the elderly ^3, 5^. Vitamin D has an essential part in metabolism regulation and has a significant role in the pathogenesis of hypertension ^4^. Since Vitamin D can inhibit renin transcription, maintain parathyroid hormone balance, vasodilation blood vessels, and reduce sympathetic nerve activity ^3^, it is reasonable that hypovitaminosis D is strongly associated with arterial hypertension. However, the result of meta-analyses has revealed that the relationship between serum 25(OH)D concentrations and a decrease in BP is inconsistent. Qi *et al*. (2017) reported that low serum 25(OH)D concentrations are not significantly associated with a risk of hypertension ^6^. In contrast, other studies demonstrated a significant relationship between low serum 25(OH)D concentrations and hypertension ^3, 7^. Another meta-analysis also proved that the serum level of 25(OH)D was significantly associated with the risk of incident hypertension on the general population ^3^.

The elderly is an age group susceptible to deficiency of this fat-soluble vitamin. Skin aging reduces 7-dehydrocholesterol production to 75%, which is known to play a key role as the main source of vitamin D in the human body ^8^. The impaired eating ability in the elderly may also contribute to low concentrations of vitamin D. Therefore, vitamin D deficiency is often associated with various geriatric syndromes. Low concentrations of vitamin D affects the activity of endocrine hormones as in sufferers of diabetes mellitus type 2 (T2DM) and cardiovascular functions, such as coronary artery disease, heart failure, stroke, and hypertension ^9^. A recent meta-analysis in individuals with vitamin D deficiency showed oral vitamin D3 reduces both systolic BP (SBP) and diastolic BP (DBP) in individuals with hypertension and decreases SBP in individuals above 50 years. In contrast, another study has revealed that in younger women, there is a strong association between high serum 25(OH)D concentrations and the risk of hypertension ^3^. Since the elderly, defined as individuals of more than 60 years of age, have a high risk of vitamin D deficiency and suffer hypertension ^1, 2, 10^, it is important to provide a meta-analysis of randomized controlled trials gathering the evidence of the effects of vitamin D supplementation compared to placebo on serum 25(OH)D concentrations and BP, specifically in the elderly population.

## Methods

### Data source and study selection

A comprehensive search was performed following the Preferred Reporting Items for Systematic Reviews and Meta-analyses (PRISMA) statement ^11^. All authors searched independently correlated studies in multiple electronic database including, PubMed, ClinicalTrials.gov, and the Cochrane Library from inception until 29 ^th^ March 2019 using a combination of keywords and subject headings. The search strategies used on PubMed and Cochrane Library using the following keywords: (vitamin D) AND ((blood pressure) OR (hypertension)) AND (elderly). While in clinicaltrials.gov, the terms used were “hypertension”, “blood pressure”, and “vitamin D”. Further relevant articles were then obtained using manually searching the references of retrieved articles.

### Eligibility criteria

All titles and abstracts were screened using Mendeley reference software, and duplications were removed manually. Full text of relevant articles were examined for eligibility criteria. The inclusion criteria were as follows: randomized controlled trial (RCT) design; participants with average age > 60 years; primary outcomes SBP change, DBP change, and serum 25(OH)D level change; and only vitamin D (cholecalciferol) intervention. The exclusion criteria were as follows: nonrandomized study; no full text available; the study control group was not placebo; outcomes relevant to our interest not reported; intervention with combined vitamin D and other nutrients supplementation; and non-English full text.

### Data synthesis and analysis

Data from each included article were extracted by three investigators (F.F., C.F., N.Y.) by utilizing a piloted form. If there is any disagreement between the authors, the final decision was made by discussion and majority vote. The following data were extracted: year of publication, year of study, geographic location, sample size, health status of participants, mean age, intervention dose, duration of the study, mean and standard deviation (SD) of serum 25(OH)D concentrations, SBP, and DBP in both intervention and placebo groups at the baseline and at the end of study, and changes from the baseline.

Each RCT’s quality was evaluated using the risk of bias tools developed by Cochrane collaboration evaluating six domains. We assessed the selection bias by evaluating the study description on the method of the randomization, the method of allocation concealment, and evaluated if there is difference in baseline between the two groups. Performance and detection bias were evaluated by finding a description about the blinding method. Attrition bias was assessed by calculating the number of participants that withdrew from the study. Reporting bias and other bias were then evaluated if there found any concern not addressed in the other domain ^12^.

The continuous data were presented as mean difference (MD) and SD. Where the change in mean (Δ Mean) was not available, we calculate the change by subtracting post intervention outcome with the baseline data. When a study did not report enough information of the change on SD (ΔSD), we calculated the data imputation applying the formula for imputing SD from baseline ^13^:

corr = ( *SDbaseline*2 + *SDpost*2 - *SDchange*2)/(2 × *SDbaseline* × *SDpost*)

ΔSD was then calculated as:

$$\Delta SD=\sqrt{\left({SD_{baseline}}^{2}+{SD_{post}}^{2}\;–\;2\;\times \;corr\;\times \;SD_{baseline}\times SD_{post}\right)}$$

To calculate the estimated effect size on MD, we used random-effect model if there was heterogeneity found using X ^2^ test and I ^2^ test ^14^. p value of <0.10 dan I ^2^ > 50% were considered high. Otherwise, the fixed-effects Mantel–Haenszel model was used. We performed univariate meta-regression analyses to evaluate differences in the continuous outcome variable. Analyses of subgroups were conducted to assess predefined sources of heterogeneity. Dose of supplementation, duration of the study, treatment regimen, hypertension, and vitamin D status were considered as sources of heterogeneity. We assessed publication bias by visual assessment on graphical funnel plots with Egger’s regression test of asymmetry ^15^.

All statistical analyses were performed using STATA 16.0 (STATA Corporation). P value < 0.05 was considered statistically significant.

## Results

### Study characteristics

**Figure 1.**
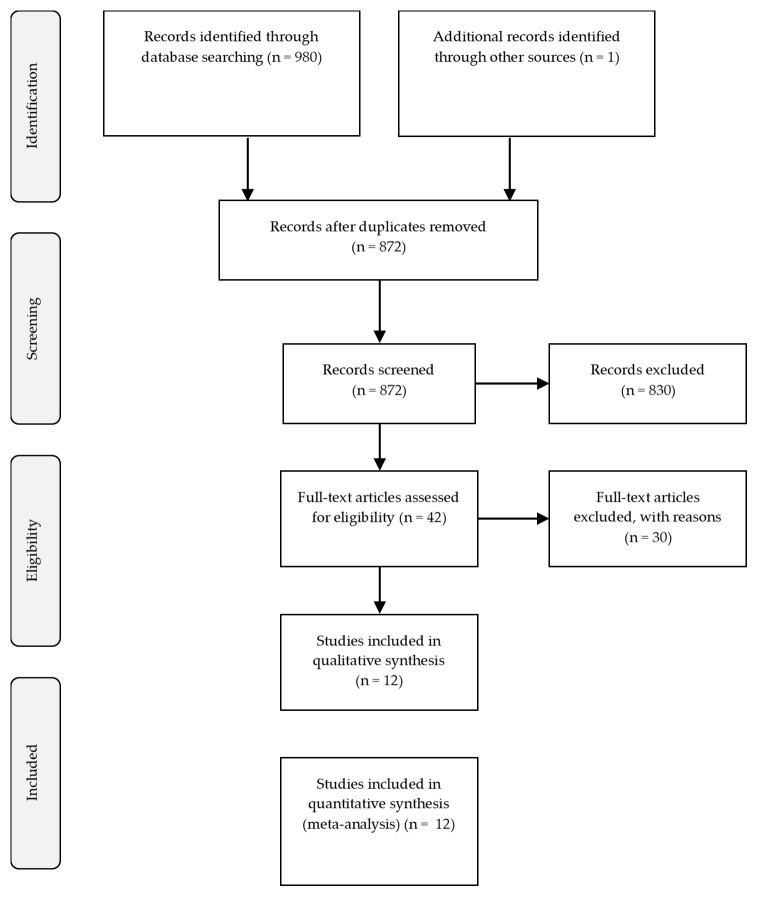
PRISMA flowchart. PRISMA, Preferred Reporting Items for Systematic Reviews and Meta-analyses.

Figure 1 presents the flowchart of this study. We screened 980 articles. Of those, 28 were excluded because of duplicate publication, and 42 articles were assessed for eligibility criteria. Of those, 30 were not eligible to be included. Finally, 12 RCTs ^16– 27^ were included in the quantitative synthesis. The quality assessment demonstrated that almost all of the included studies has a low risk of bias. The results for quality assessment was summarized in *Extended data:* Figure S1 ^28^.

Table 1 summarizes the characteristics of the included RCTs. The RCTs were conducted in different continents: Asia ^23^, Europe ^19– 22, 25– 27^, America ^17^, and Oceania ^18^. All were placebo controlled and published in English. The mean age of the participants was 65.5 years with differing health conditions. Only three RCTs included participants without certain medical criteria but with some conditions that indicated vitamin D deficiency, including postmenopausal women ^16, 17^ and those taking vitamin D supplements < 400 IU ^19^. In addition, several RCTs targeted conditions related to blood sugar concentrations, such as T2DM ^21, 26^ and prediabetes ^24^. Hypertension patients were also the subjects of several RCTs, which focused on isolated systole hypertension (ISH) ^22^, arterial hypertension ^20, 25^, and essential hypertension ^23^.

**Table 1.** Supplementation of vitamin D on 25(OH)D serum and blood pressure in the elderly.

Authors	Countries, continent	*n*	Subject condition	Average age (years)	Before intervention		Dose	Time (wk)	After intervention	
					25(OH)D ng/mL	BP (mmHg)			25(OH)D ng/mL	BP (mmHg)
Chen, *et al.* ^23^ (2014)	China, Asia	126	-HT grade I-II -Consume nifedipine 30 mg/d	62.5±9.1	19.4±11.6	SBP: 132.1±9.4 DBP: 75.1±9.1	2,000 IU/day	24	34.1±12.2 *	∆SBP: -6.2 * ∆DBP: -4.2 *
Wood, *et al.* ^16^ (2012)	UK, Europe	265	Healthy post-menopausal women	63.5±1.9	13.12±5.2	SBP: 128.2±13.8 DBP: 77.7±7.3	400 IU/day	48	46.1±5.2 *	∆SBP: -2.2 ∆DBP: -2.5
				64.1±2.3	13±5.5	SBP: 129.2±15.6 DBP: 76.9±8.1	1,000 IU/day	48	55.9±5.5 *	∆SBP: -1.5 ∆DBP: -0.9
Witham, *et al.* ^22^ (2013)	UK, Europe	159	-ISH -25(OH)D concentrations <30ng/mL	76.9±4.8	18±6.0	SBP: 163±11 DBP: 78±7	100,000 IU/3 month	48	26±6.0 *	SBP: 163±18 DBP: 78±9
Witham, *et al.* ^21^ (2010)	UK, Europe	61	-T2DM -25(OH)D serum <40 ng/mL	65.3±11.1	16.2±5.6	SBP: 149.6±24.0 DBP: 81.7±12.4	100,000 IU once	32	25.2±8	SBP: 141.4±16.6 * DBP: 77.1±11.7
							100,000 IU once	64	23.6±7.2	SBP: 144.6±20.4 DBP: 79.6±11.9
				63.3±9.6	19.2±8.4	SBP: 145.1±25.0 DBP: 80.7±14.3	200,000 IU once	32	31.7±12.4 *	SBP: 136.8±12.9 * DBP:74.4±9.8
							200,000 IU once	64	30.5±12 *	SBP: 139.5±15.4 DBP: 77.6±11.7
Sollid, *et al.* ^24^ (2014)	Norway, Europe	511	Prediabetes	62.1±8.7	24±8.8	SBP: 135.4±16.8 DBP: 83.2±10.1	20,000 IU/wk	48	42.4±9.7 *	∆SBP: -2.9±13.7 ∆DBP: -4.6±8.9
Gepner, *et al.* ^17^ (2012)	Wisconsin, American	114	-Post-menopausal women -25(OH)D concentrations >10 and <60 ng/mL	64.1±3	30.3±10.7	SBP: 122.3±13.1 DBP: 72.5±7.6	2,500 IU/day	16	46.0±9.3 *	∆SBP: -0.3±8.4 ∆DBP: -0.7±5.1
Pilz, *et al.* ^25^ (2015)	Austria, Europe	188	-Arterial HT -25(OH)D serum <30 ng/mL -Ongoing antihypertensive treatment	60.1±11.3	22.0±5.5	SBP: 131.4±8.1 DBP: 78.1±7.5	2,800 IU/day	32	36.2±7.3 *	SBP: 130.3±9.3 DBP: 77.8±8.2
Sugden, *et al.* ^26^ (2008)	Scotland, Europe	34	-T2DM -25(OH)D serum <20ng/mL -6 weeks stable medication	64.9±10.3	16.1±4.1	SBP: 145 ± 9.2 DBP: 82 ± 10.5	100,000 IU once	32	25.3±6.7 *	∆SBP: -7.3±11.8 * ∆DBP: -2.2±8.6
Larsen, *et al.* ^20^ 2012)	Denmark, Europe	112	-Arterial HT -Unchanged medications during study	61±10	23.0±9.0	SBP: 132 ± 10 DBP: 77 ± 6	3,000 IU/day	80	44.0±9.0 *	SBP: 130±11 DBP: 76±7
					<32					∆SBP: -4 * ∆DBP: -3 *
Stricker, *et al.* ^27^ (2012)	Switzerland, Europe	76	-Chronic Peripheral Arterial Disease -25(OH)D serum <30 ng/mL	72.9±8.7	16.3±6.7	SBP: 133±18.5 DBP: 73±8.2	100,000 IU once	4	24.3±6.2 *	SBP: 136±18.7 DBP: 73±8.1
Sluyter, *et al.* ^18^ (2017)	New Zealand, Oceania	517	-Both men and women aged 50–84 years	63.3±8.6	11.1±3.2	SBP: 137.4±16.8 DBP: 78.9±10.7	initiation 200,000 IU, next 100,000 IU/month	48	23.2 *	SBP: 128.9±16.1 DBP: 73.7±9.9
Tomson, *et al.* ^19^ (2017)	UK, Europe	305	-Participants aged minimum 65 years -Not taking >400 IU vitamin D daily	71	15.7	SBP: 132.7±21.1 DBP: 78±11.3	4,000 IU/day	48	43.1±0.8 *	SBP: 132.5±1.43 DBP: 77.2±0.9
				72		SBP: 131.8±17.1 DBP: 76.6±10.3	2,000 IU/day	48	32.1±0.8 *	SBP: 131.8±1.51 DBP: 76.6±0.96

HT, hypertension; 25(OH)D, 25-hydroxyvitamin D; BP, blood pressure; SBP, systolic blood pressure; DBP, diastolic blood pressure; ∆SBP/DBP, changes of SBP/DBP; wk, weeks; ISH, isolated systole hypertension; T2DM; type 2 diabetes mellitus; *, significant

The type of hypertension affected the baseline BP of the participants. The BP varied; some participants had hypertension, whereas others had normal BP. SBP in all participants ranged from 109.2 to 174 mmHg, whereas DBP ranged from 64.8 to 95 mmHg. High SBP usually occurred in ISH and was most commonly found in the elderly. However, on average, in each RCT, the participant’s BP was categorized as prehypertension or hypertension (>120/80 mmHg). Hypertension and diabetes experienced by the elderly made it difficult for the participants to be excluded on the basis of medications. Therefore, some of the RCTs had inclusion criteria that required participants not to change their medical treatment throughout the study duration ^20, 26, 27^, because the use of different drugs affects vitamin D intervention. In their study at one of the general hospitals in Beijing, China, Chen *et al*. (2014) included participants who were taking 30 mg/dL of nifedipine ^23^. Another factor that may influence the effectiveness of vitamin D supplementation was baseline 25(OH)D concentrations before the intervention. Some of the RCTs had set serum 25(OH)D limits to <20 ^26^, 30 ^22, 25, 27^, 40 ^22^, or 60 ng/mL ^17^; all these values indicate deficiency in vitamin D.

### 25(OH)D concentrations change

**Figure 2.**
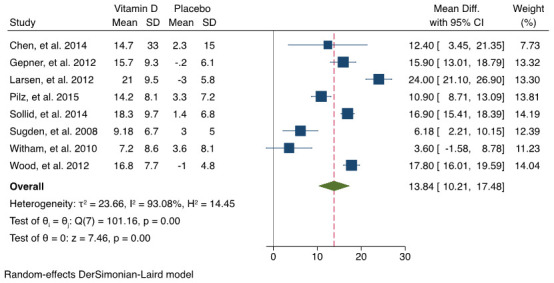
Forest plot of 25-hydroxyvitamin D changes from the baseline. The overall effect size estimate is represented by the red dashed line. SD, standard deviation; CI, confidence interval.

Table 1 shows that most participants revealed baseline data of mean 25(OH)D concentrations <20 ng/mL, and the maximum was 30 ng/mL ^17^. Eight RCTs evaluated changes of 25(OH)D concentrations after giving vitamin D intervention. The 1293 participants were divided into treatment (n = 641) and control groups (n = 652). Pooling data revealed that the vitamin D group had a significant higher serum 25(OH)D concentrations compared to the control group (MD = 13.84; 95% CI = 10.21–17.47; P < 0.0001). We observed heterogeneity among the RCTs (I ^2^ = 93%), so we selected a random-effects model ( Figure 2).

### BP change

**Figure 3.**
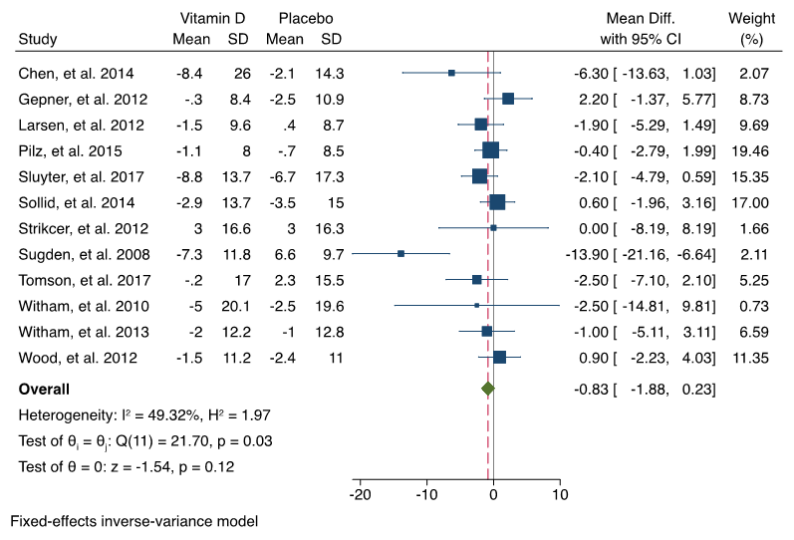
Forest plot of systolic blood pressure changes from the baseline. The overall effect size estimate is represented by the red dashed line. CI, confidence interval; SD, standard deviation.

**Figure 4.**
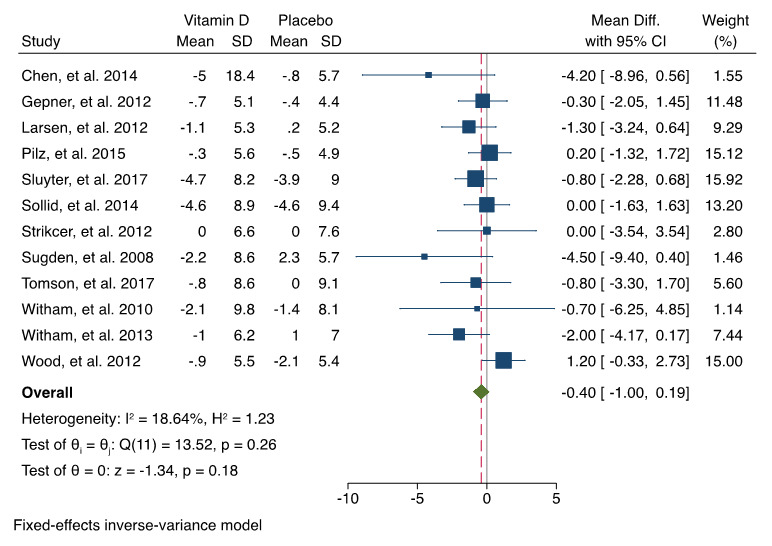
Forest plot of diastolic blood pressure changes from the baseline. The overall effect size estimate is represented by the red dashed line. CI, confidence interval; SD, standard deviation.

**Figure 5.**
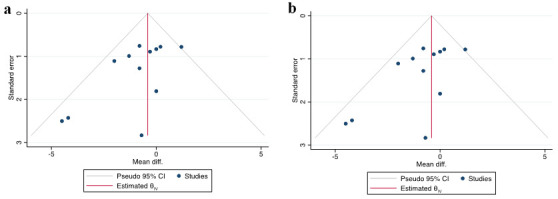
Funnel plot assessing publication bias for the effect of ( **a**) systolic blood pressure (BP) change and ( **b**) diastolic BP change.

The change of SBP and DBP was synthetized from 12 RCTS. We did not observe heterogeneity among the RCTs (I ^2^ < 50%), so we selected a fixed-effects Mantel–Haenszel model. Pooled analysis revealed that overall, the SBP change (MD = –0.83; 95% CI = –1.88 to 0.23; P = 0.12) and DBP changes (MD = 0.40; 95% CI = –1.00–0.19; P = 0.18) in vitamin D group were not significant compared with the control group. The effects of vitamin D on SBP and DBP were summarized as forest plots presented in Figure 3 and Figure 4. The funnel plots of SBP change and DBP change are summarized in Figure 5. Our analysis showed that there was no publication bias when examining funnel plots and the result of Egger’s test of asymmetry for SBP change and DBP change ( *p* = 0.158; *p*= 0.069; respectively).

**Figure 6.**
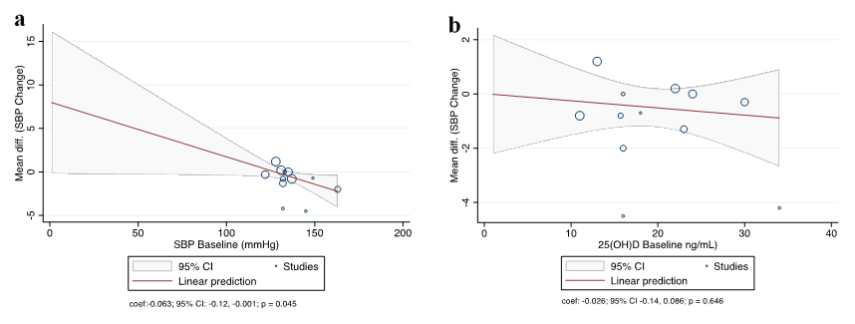
Bubble plot of univariate random-effects meta-regression. ( **a**) Participant blood pressure baseline and the mean difference (MD) in systolic blood pressure (SBP) change; ( **b**) Participant 25(OH)D baseline and the MD in SBP change. Each circle characterizes a study and the size of the circle reflects the influence of that study on the model. The regression prediction is represented by the solid line.

Table 2 and Table 3 present the pooled estimated effect size of vitamin D on the change of SBP and DBP, on the basis of BP baseline, vitamin D status baseline, intervention dose, treatment duration, and treatment regimen. Our analysis indicate that vitamin D supplementation had no significant influence on SBP and DBP changes on the basis of dose, duration, and treatment regimen. However, subgroup analysis revealed a marginal trend toward significance in terms of DBP changes with treatment duration ≤ 6 months (MD = –0.82; 95% CI = –1.66 to –0.02; P = 0.05). Subgroup analysis by hypertensive and deficiency of vitamin D status indicated that vitamin D supplementation could significantly reduce SBP (MD = –4.01; 95% CI = –7.45 to –0.57; P = 0.02 and MD = –1.91; 95% CI = –3.48 to –0.34; P = 0.02, respectively). However, we found a significant difference in DBP changes only in the hypertensive subgroup (MD = –2.22; 95% CI = –4.1 to –0.34; P = 0.02) ( Table 2 and Table 3). The forest plot of each subgroup analysis is available as *Extended data:* Figure S2 ^28^. Our study provided enough observations to conduct univariate meta-regression, summarized in Table 2 and Table 3. The result for SBP change was presented as bubble plots in Figure 6.

**Table 2.** Subgroup analysis of SBP changes.

	Trials	MD (95%CI)	*p*-Value	*I ^2^ (%)*
**BP baseline**				
Hypertension baseline	3	-4.01 (-7.45,-0.57)	0.02*	78
Normal baseline	9	-0.50 (-1.61,0.61)	0.38	9
**Baseline Vitamin D status**				
Vitamin D deficiency	8	-1.91(-3.48,-0.34)	0.02*	55
Normal baseline	4	0.06 (-1.37,1.48)	0.93	0
**Duration**				
≤ 6 months	8	-1.13 (-2.61,0.35)	0.13	61
> 6months	4	-0.51 (-2.02,0.99)	0.51	16
**Intervention dose**				
≤ 2000 IU/d	2	-0.21 (-3.09,2.67)	0.88	68
> 2000 IU/d	6	-0.47 (-2.07,1.14)	0.57	16
**Regiment treatment**				
Daily	6	-0.41 (-1.81,1)	0.57	26
Intermittent	6	-1.38 (-2.98,0.22)	0.09	65

SBP, systolic blood pressure; BP, blood pressure; MD, mean difference; CI, confidence interval.

**Table 3.** Subgroup analysis of DBP changes.

	Trials	MD (95%CI)	*p*-Value	*I ^2^ (%)*
**BP baseline**				
Hypertension baseline	3	-2.22 (-4.1,-0.34)	0.02*	0
Normal baseline	9	-0.20 (-0.83,0.42)	0.52	4
**Baseline Vitamin D** **status**				
Vitamin D deficiency	4	-0.55 (-1.38,0.28)	0.20	40
Normal baseline	8	-0.25 (-1.1,0.59)	0.56	0
**Duration**				
≤ 6 months	8	-0.82 (-1.66,0.02)	0.05	9
> 6 months	4	-0.41 (-83,0.86)	0.97	22
**Intervention dose**				
≤ 2000 IU/d	2	0.69 (-0.76,2.15)	0.35	78
> 2000 IU/d	6	-0.41 (-1.33,0.51)	0.38	0
**Regimen treatment**				
Daily	6	-0.09 (-0.87,0.68)	0.81	34
Intermittent	6	-0.83 (-1.75,0.08)	0.07	0

DBP, diastolic blood pressure; BP, blood pressure; MD, mean difference; CI, confidence interval.

## Discussion

### Effects of vitamin D on serum 25(OH)D concentrations

Serum 25(OH)D concentrations has a major role as a marker for determining vitamin D status in humans. As mentioned before, most vitamin D circulates in human body in the form of 25(OH)D. This is a result of vitamin D metabolism from the skin and vitamin D intake and binds to vitamin D-binding protein, which has a half-life of 2–3 weeks. The clinical practice guidelines issued by the Institute of Endocrinology has defined vitamin D deficiency as levels of 25(OH)D below 20 ng/mL ^29^. In addition, the average normal value for serum 25(OH)D concentrations for all ages is 30 ng/mL, whereas in the elderly it is >20 ng/mL or 50 nmol/L ^30^.

Deficiency in vitamin D could be caused by physiological and pathological factors in the elderly. One of the most common physiological factors is decreasing pre-vitamin D production in the skin. The reasons are that compared with young adults, the skin’s capacity to produce vitamin D decreases by 75% at 70 years ^29, 31^. In addition, the elderly have a tendency to wear closed clothing for fear of flu, thus causing minimal exposure to ultraviolet B rays ^9^. Their food intake decreases because of a decrease in chewing ability and financial conditions ^30^. Also, decreased calcium absorption results in impaired vitamin D metabolism and decreased kidney function ^29– 34^. Pathological factors are related to organs that play a role in the digestion and metabolism of vitamin D. Decreased bioavailability in the digestive tract (malabsorption due to disease) inhibits vitamin D metabolism. Patients with liver disease can suffer from vitamin D hydroxylation disorders. Kidney pathologies, such as nephrotic syndrome and chronic kidney disease, reduce renal activation ^34^. However, such problems were amongst the exclusion criteria.

One of the main findings of the present meta-analysis was vitamin D supplementation has significant effect on serum 25(OH)D concentrations among the elderly. It increases serum 25(OH)D concentrations in people that are older than 60 years old. Almost all studies included have revealed a significant increase in serum 25(OH)D concentrations from the baseline. The contradictory result was shown by Witham *et al.* (2010) that inconsistent with a previous study by Sugden *et al.* (2008), which has revealed a significant difference between the treatment and control groups with same doses and duration ^24^. Another study has reported increasing serum 25(OH)D concentrations at follow-up in the vitamin D group, with no change in the placebo group ^18^. The relationship between serum 25(OH)D concentrations and a decrease in BP is still debatable. A meta-analysis of observational cross-sectional and prospective studies on general populations has proven the relationship between serum 25(OH)D concentrations and the risk of incident hypertension ^3^. However, a newer meta-analysis showed oral vitamin D3 has no significant effect on blood pressure in individuals with vitamin D deficiency ^5^.

### Effects of vitamin D on BP

This study included research from four different continents. However, characteristically there are no specific differences for each continent. The results were random and more relevant to the baseline data and effect of the RCT itself. The present study provides evidence that although the supplementation could increase serum 25(OH)D concentrations, there was no significant difference in SBP and DBP changes compared with the control group. It means that the increasing serum 25(OH)D concentrations were not followed by decreasing BP among elderly. Several studies have revealed not only a relationship between an increase in serum 25(OH)D concentrations and a decrease in BP after vitamin D supplementation but also a significant change in other conditions, such as parathyroid hormone, serum calcium, renin, and angiotensin II concentrations ^16, 20, 23^, indicating that vitamin D regulates a decrease in BP through various mechanisms. The effect of vitamin D supplementation on a decrease in BP is inconsistent in several studies. Some studies have reported that vitamin D supplementation can reduce BP, although only SBP, so vitamin D supplementation can be a supportive therapy for hypertension ^8, 21, 26^. Similar to our result, a meta-analysis by Golzarand *et al*. has revealed that vitamin D supplementation is only associated with an increase in serum 25(OH)D concentrations, not SBP or DBP ^35^. Meanwhile, according to Chen *et al.* (2014), vitamin D supplementation that complements 30 mg/dL nifedipine in patients with grade I or II essential hypertension can reduce SBP or DBP ^23^. They were the first to look at the effectiveness of the interaction between vitamin D and specific drugs in contrast to other studies that only provide inclusion criteria in the form of no change in medication consumption.

Several meta-analyses have been conducted to evaluate the association between vitamin D and BP. The findings of the meta-analysis of observational studies on general populations have confirmed the association between vitamin D status and the risk of incident hypertension ^3, 7, 8^. However, the associations are not proof of causality, so placebo-controlled RCTs are required in order to prove the effects of vitamin D on BP. Previous RCTs and meta-analyses have revealed that vitamin D might be beneficial in lowering BP, especially in vitamin D-deficient patients with hypertension, which is similar to our results ^4, 18^. In contrast to our findings, Ke *et al.* (2015) reported no increased risk of hypertension in the elderly or in vitamin D-deficient participants; however, their research involved a prospective study design ^3^. The other important finding in our study was significant differences in SBP and DBP changes among the hypertensive subgroup. Previous meta-analyses have also revealed a significant effect of vitamin D on BP in patients with hypertension at the baseline and no significant decrease in normotensive patients at the baseline ^5, 36^. In contrast to our findings, a meta-analysis by Golzarand *et al*. (2016) showed that vitamin D showed hypotensive effects in both healthy and hypertensive subjects ^35^. To the best of our knowledge, there has been no research that suggests that there are differences in the metabolism of vitamin D in the elderly with hypertension and norm tension, except secondary hypertension associated with kidney organs ^37^. Increased BP also occurs in arterial hypertension patients who have vitamin D deficiency. Patients with deficiency in vitamin D may acquire the effects of vitamin D supplementation ^20^. However, administering vitamin D to participants who meet the same criteria can give zero results with regard to a decrease in BP because of the shorter time of administration (only 8 weeks with almost the same dose) ^25^.In addition, an updated meta-analysis’s results were similar to our study in that subgroup analysis showed vitamin D supplementation may reduce SBP and DBP in patients with low 25(OH)D concentrations and hypertension ^5^.

In addition to hypertension patients, T2DM patients also exhibit a decrease in BP, although only SBP, after a high dose of vitamin D supplementation ^21, 26^. Again, serum 25(OH)D concentrations also contributed to the effects of vitamin D supplementation on lowering BP. Pre diabetic patients reveal absolutely no effect of the same dose of vitamin D on BP because they are not vitamin D deficient ^24^. Most of the RCTs included in this study reveal an insignificant decrease in BP after given a dose supplementation of vitamin D. In the case of ISH, which is common among the elderly, vitamin D supplementation is less effective. The reason is probably because vitamin D cannot decrease blood vessel stiffness and is effective only during the early stages of the disease. Circulating renin concentrations are not raised in the elderly and not the other way around as renin raises the BP and is suppressed by vitamin D ^22^. Other studies have revealed no effect of vitamin D supplementation on lowering BP in postmenopausal women ^17^, chronic peripheral arterial disease patients ^20^, and the elderly without certain medical conditions ^16, 18, 19^. The actual conditions suffered by the elderly due to multiple chronic diseases and therefore multiple drugs should be considered as an important aspect that may influence the effects of vitamin D on BP.

Our study revealed that vitamin D only reduces blood pressure on hypertensive elderly. Similar to our findings, most studies identified lower BP reduction by dietary or drug interventions in hypertensive people rather than normotensive populations ^38– 40^. Our analysis showed that Vitamin D supplementation could significantly reduce SBP by -4.01 mm Hg (95% Cl: -7.45 to -0.57) and DBP by -2.22 mm Hg (-4.1 to -0.34) for hypertensive elderly. The observed reduction of BP is similar to a meta-analysis by Filippini 2017 showing potassium supplementation decreased SBP of -4.48mm Hg (95% CI -3.07 to -5.90) and DBP of -2.96 mmHg (-1.10 to -4.82) ^41^. Moreover, the present findings showed higher BP reduction than calcium supplementation that reduced SBP by −1.86 mm Hg (95% CI: −2.91 to −0.81) and DBP by −0.99 mm Hg (−1.61 to −0.37) ^42^. However, our result showed lower BP reduction than a meta-regression analysis of sodium reduction (SR) that showed strong evidence of a linear dose-response relation between SR and BP among hypertensive individuals. For SBP, the dose-response relation was -7.7 mm Hg/100 mmol SR (95% CI: -10.4 to -5.0), and for DBP it was -3.0 mm Hg/100 mmol SR (95% CI: -4.6 to -1.4) ^43^. Furthermore, the DASH diet that was originally developed to contain food high in magnesium, potassium, calcium and low sodium showed significantly decreased SBP by *-*5.2 mmHg (95% CI, *-*7.0 to *-*3.4) and DBP by *-*2.60 mmHg ( *-*3.50 to *-*1.70) ^44^. In contrast, a non-significant reduction in systolic BP was demonstrated by calcium and vitamin D co-supplementation ^45^.

A meta-analysis of 34 RCTs demonstrated that SBP/DBP reduction by -10/-5 mmHg significantly reduces negative health outcomes ^46^. Another study by Andersen *et* al. reported 9.2 mmHg reduction in SBP might represent improvements in treatment. Even though our findings showed a lower BP reduction than the aforementioned studies, this result might be due to the elderly were reported as being treated less successfully than young and middle-aged individuals ^47^. Moreover, this assessment suggests that the 4.01 mmHg reduction in SBP might be considered as clinically meaningful since a meta-analysis on the effects of dietary interventions showed clinically significant BP reduction with the average net change in SBP and DBP of -3.31 mmHg and -2.24 mmHg, respectively ^38^.

Searching articles until the end of March 2019, our study provides the most up-to-date meta-analysis and strongly supports that vitamin D supplementation significantly decreases BP in the elderly, specifically with elevated BP and deficiency in vitamin D. To the best our knowledge, this is the first study that analyses the effects of vitamin D on serum 25(OH)D value in elderly people for both vitamin D status and effects on blood pressure. The newest meta-analysis has revealed that vitamin D has no significant effect on BP in vitamin D-deficient people; it reduces SBP in vitamin D-deficient people older than 50 years and in people with both vitamin D deficiency and hypertension ^5^. However, a previous meta-analysis involved subjects between 18 and 74 years old and serum 25-OHD are lower than 20ng/mL, and subgroup analysis used criteria older than 50 years old. Meanwhile, our study involved subjects with the mean age more than 60 years, according to WHO definition for elderly ^10^. However, this study had a few limitations. First, although we conducted a systematic review of peer-reviewed research, we did not include agency reports, dissertations, and conference proceedings. Second, we included only English-language RCTs. Third, the RCTs were heterogeneous with respect to demographic characteristics of the participants, the duration, supplemental doses, and treatments for hypertension

## Conclusions

Vitamin D deficiency is prevalent among the elderly, and vitamin D supplementation significantly increases serum 25(OH)D concentrations. The use of vitamin D supplementation appears to be beneficial in lowering BP, specifically in the elderly with hypertension and vitamin D deficiency. We recommend vitamin D supplementation for elderly individuals with hypertension and serum 25(OH) D concentrations below the target values. The actual conditions suffered by the elderly because of multiple chronic diseases and therefore multiple drugs should be considered as important factors that influence the effects of vitamin D on BP. Future studies with homogenous treatment duration, dose intervention, and treatment regimens need to be carried out to identify optimal treatment regimens for hypertension in the elderly that may need to include correction of vitamin D deficiency.

## Data availability

### Underlying data

All data underlying the results are available as part of the article and no additional source data are required.

### Extended data

Open Science Framework: Extended data for “Effects of vitamin D supplementation on 25(OH)D concentrations and blood pressure in the elderly: a systematic review and meta-analysis.” http://doi.org/10.17605/OSF.IO/EXF26 ^28^.

This project contains the following extended data:

- Spreadsheets in .sav format containing data for supplementation efficacy outcomes in 25(OH) concentrations, systolic blood pressure, and diastolic blood pressure.
- Supplementary figure in .doc format containing: Figure S1: results for quality assessment, Figure S2: forest plot of each subgroup analysis, Figure S3: Funnel plot and egger test results.

### Reporting guidelines

Open Science Framework: Extended data for “Effects of vitamin D supplementation on 25(OH)D concentrations and blood pressure in the elderly: a systematic review and meta-analysis.” http://doi.org/10.17605/OSF.IO/EXF26 ^28^.

Data are available under the terms of the Creative Commons Zero "No rights reserved" data waiver (CC0 1.0 Public domain dedication).
