# Supplementary material for: Effects of vitamin D supplementation on 25(OH)D concentrations and blood pressure in the elderly: a systematic review and meta-analysis
Source: F1000Res. 2020 Sep 3;9:633. Originally published 2020 Jun 22. [Version 3] doi: 10.12688/f1000research.24623.3 (PMC7489275; doi:10.12688/f1000research.24623.3)
Supplement: Peer review history

# Peer review: Reviewer response for version 3

**DOI:** 10.5256/f1000research.29059.r70734

**Published:** 2020-09-11

**Authors:** Alexandre S. Silva

**Affiliations:** 1 Postgraduate Program in Nutrition Sciences-Postgraduate Program in Physical Education, Federal University of Paraíba, João Pessoa, Brazil

In the previous review round, I had said that both the review and the meta-analysis were well structured, but I suggested minor changes to the text to make the manuscript more convincing. The authors responded satisfactorily to all suggestions. The changes made are indicated below:

1. As suggested in the introduction, the authors presented the biological rationale between hypovitaminosis D and hypertension.
2. The authors detailed the search terms for other bases besides Medline / Pubmed.
3. In the discussion, the authors quantified the hypotensive effect of vitamin D and presented an opinion, based on the evidence, as to the relevance of the use of vitamin D for the treatment of arterial hypertension.

Are the rationale for, and objectives of, the Systematic Review clearly stated?

Partly

Is the statistical analysis and its interpretation appropriate?

Yes

Are sufficient details of the methods and analysis provided to allow replication by others?

Partly

Are the conclusions drawn adequately supported by the results presented in the review?

Yes

Reviewer Expertise:

Nutrition, exercise and Hypertension; Nutrition, exercise and weight loss; Fresh food as ergogenic aid for athletes; nut

I confirm that I have read this submission and believe that I have an appropriate level of expertise to confirm that it is of an acceptable scientific standard.

# Peer review: Reviewer response for version 3

**DOI:** 10.5256/f1000research.29059.r70735

**Published:** 2020-09-04

**Authors:** Barbara J. Boucher

**Affiliations:** 1 The Blizard Institute, Barts & The London School of Medicine & Dentistry, Queen Mary University of London, London, UK

The amendments made do provide improved clarity of this text and I have no further matters of importance to raise.

Are the rationale for, and objectives of, the Systematic Review clearly stated?

Yes

Is the statistical analysis and its interpretation appropriate?

I cannot comment. A qualified statistician is required.

Are sufficient details of the methods and analysis provided to allow replication by others?

Yes

Are the conclusions drawn adequately supported by the results presented in the review?

Partly

Reviewer Expertise:

diabetes, metabolism, vitamin d and betel quid chewing.

I confirm that I have read this submission and believe that I have an appropriate level of expertise to confirm that it is of an acceptable scientific standard.

# Peer review: Reviewer response for version 2

**DOI:** 10.5256/f1000research.27711.r69387

**Published:** 2020-08-18

**Authors:** Barbara J. Boucher

**Affiliations:** 1 The Blizard Institute, Barts & The London School of Medicine & Dentistry, Queen Mary University of London, London, UK

This review and meta-analysis of previous studies on the effects of vitamin D supplementation on vitamin D status and on blood pressure in the elderly is a topic of importance for population health since hypertension and vitamin D deficiency are both so common in elderly people globally,  including isolated systolic hypertension. This manuscript has already been assessed by earlier peer review as being appropriate in design and analytical methodology. The present comments, therefore, are mainly directed at presentation, readability and the additional information requested by the previous reviewers. It is unfortunate that only 8 of the many RCTs considered for this study were suitable for meta-analysis. The use of Individual Participant Data, if obtainable, could be more revealing on the question of whether supplementation is more effective for reducing BP in older people in deficiency than in repletion, as suspected in other health problems and as the present data suggests. This would be a valuable follow-on study if it were to be possible.

**General comments:**

1. The English language usage is often awkward, which distracts from the readability of the text. Simple editing by a native English language speaker with a scientific/medical background would greatly improve the readability of the text throughout.
2. Vitamin D was not measured in any studies analysed, but 25-hydroxyvitamin D [25OHD], and this term should be used throughout. Lab data reports ‘concentrations’ not ‘levels’ and this term should be used while ‘levels’ is good for cut-offs and definitions.

**Specific comments:**

- Table 2, the heading ‘vitamin D’ would be clearer saying ‘baseline vitamin D status’.
- Page 10, column 1, line 2, do you mean decreases by 75% or decreases by 25% - please clarify; line 3, ..have..; 2 ^nd^ column, line 6, do you mean ‘…reduce renal activation, [though not that in other target tissues], but such problems were amongst the exclusion criteria’?
- Page 10, 2 ^nd^ column, para 2, the last sentence reads as if this is the 1 ^st^ study to assess the effects of supplementation on serum 25OHD values but this cannot be true either for this submission or for the ‘newer’ meta-analysis mentioned in the previous sentence that needs a reference. These comments should be corrected - maybe you mean  ‘...in elderly people for both vitamin D status and effects on blood pressure’?
- Page 11, 1 ^st^ column, line 15, 'adjunctive' might be a better word than 'supportive'; Para 2, line 4, ‘....have confirmed the association’ might be better than proven & ‘ ….however associations are not proof of causality …..; column 2, 1 ^st^ para, lines 6/7 are obscure, this section reads as if deficiency lowers blood pressure, which is not what is meant - maybe say, re. ref 24, ‘ ...because they were not vitamin D deficient’. Re ref 22 comment, it would be better to say that circulating renin concentrations are not raised in the elderly and not the other way around as renin raises the BP and is suppressed by vitamin D, [if you are sure that this is a general finding]. Para 3, do you mean that supplemental doses were heterogeneous or that treatments for hypertension were heterogeneous, or, as I suspect, that both of these were heterogeneous? Please clarify.
- Page 12, para 1, is unclear, if you mean that studies homogeneous for duration, etc, need to be carried out, please rephrase for clarity, perhaps concluding, ‘...so as to identify optimal treatment regimes for hypertension in the elderly that may need to include correction of vitamin D deficiency’?

Are the rationale for, and objectives of, the Systematic Review clearly stated?

Yes

Is the statistical analysis and its interpretation appropriate?

I cannot comment. A qualified statistician is required.

Are sufficient details of the methods and analysis provided to allow replication by others?

Yes

Are the conclusions drawn adequately supported by the results presented in the review?

Partly

Reviewer Expertise:

diabetes, metabolism, vitamin d and betel quid chewing.

I confirm that I have read this submission and believe that I have an appropriate level of expertise to confirm that it is of an acceptable scientific standard.

# Response

**Published:** 2020-09-04

**Authors:** Niwanda Yogiswara

**Affiliations:** Airlangga University, Indonesia

We appreciate Professor Barbara Joan Boucher for her time, constructive comments, and for recommending approval of our paper. 

**The major recommendations for improvement are two:**

First, "The English language usage is often awkward, which distracts from the readability of the text. Simple editing by a native English language speaker with a scientific/medical background would greatly improve the readability of the text throughout." We have tried to revise some sentence misconstruction and also improve the English language. Every specific comment and suggestion indicated by the Reviewer has been precisely considered, and we hope satisfactorily addressed. 

Second, the Reviewer points out that the paper should use the term 'concentrations' not 'levels'. This is an important suggestion since lab data reports 'concentrations' not 'levels', we have replaced the term 'levels' with 'concentrations', except for cut-off and definitions.

Again, we thank Prof. Boucher for giving her valuable insight with very detailed comments, which we feel have significantly improved paper readability.

# Peer review: Reviewer response for version 2

**DOI:** 10.5256/f1000research.27711.r68253

**Published:** 2020-08-05

**Authors:** Alexandre S. Silva

**Affiliations:** 1 Postgraduate Program in Nutrition Sciences-Postgraduate Program in Physical Education, Federal University of Paraíba, João Pessoa, Brazil

It is a review with a well-structured meta-analysis, following Cochrane principles. Compared to other previous meta-analyzes, the present review differs in that it performed the analysis in the elderly population. Although it has been noticed that this manuscript has already been revised, I still see some small issues that can be improved, especially to ensure the replicability of the methodology. This and other questions are presented below.

 Introduction:

- Second paragraph: I suggest the authors to present the biological rationale of hypovitaminosis D with arterial hypertension (before presenting the results of some meta-analyzes).

 

 Methods:

- The authors presented the PubMed search strategy only. I suggest presenting the other strategies, so that the methodology is replicable.
- One of the exclusion criteria was: "assessment of the full text of the study not possible". This actually happened? I suggest that the authors identify these studies and present the difficulties to access this study (s). Important for the replication of the methodology.

 

 Discussion:

- I missed the clinical implications of this study. Do data provide evidence for the use of vitamin D for hypertensive elderly people? Although significant, does the observed reduction in blood pressure really have clinical relevance compared to other interventions?

Are the rationale for, and objectives of, the Systematic Review clearly stated?

Partly

Is the statistical analysis and its interpretation appropriate?

Yes

Are sufficient details of the methods and analysis provided to allow replication by others?

Partly

Are the conclusions drawn adequately supported by the results presented in the review?

Yes

Reviewer Expertise:

Nutrition, exercise and Hypertension; Nutrition, exercise and weight loss; Fresh food as ergogenic aid for athletes; nut

I confirm that I have read this submission and believe that I have an appropriate level of expertise to confirm that it is of an acceptable scientific standard, however I have significant reservations, as outlined above.

# Response

**Published:** 2020-09-04

**Authors:** Farapti Farapti

**Affiliations:** Faculty of Public Health, Indonesia

We thank the editors for facilitating the peer-review process and we appreciate Professor Silva's positive opening remarks and his expert review and suggestions for improving the paper. We have uploaded a revised copy of the manuscript. 

**The specific recommendations for improvement are four:**

First, the Reviewer suggested that the paper should “present the biological rationale of hypovitaminosis D with arterial hypertension before presenting the results of some meta-analyzes”. In the revised paper, we have now added the sentence to present the biological rationale of hypovitaminosis D with arterial hypertension in the introduction paragraph two.  

Second, the Reviewer suggested that the paper should present the strategy used in other databases. In our revision, we have added the information regarding other strategies used in Cochrane Library and Clinicaltrial.gov so that the methodology is replicable.

Third, "One of the exclusion criteria was: "assessment of the full text of the study not possible". This actually happened? I suggest that the authors identify these studies and present the difficulties to access this study (s). Important for the replication of the methodology". We are sorry that we used a phrase that leads to sentence misconstruction. The sentence "assessment of the full text of the study not possible" should be written by "no full text available." 

Fourth, "I missed the clinical implications of this study. Do data provide evidence for the use of vitamin D for hypertensive elderly people? Although significant, does the observed reduction in blood pressure really have clinical relevance compared to other interventions?"

This is an important question and we have addressed it directly in the revision. In the revised paper, we now incorporate the study's clinical implication and cite the relevant papers regarding clinical relevance compared to other intervention in the discussion section. We thank Prof. Silva for calling our attention to these ideas.

Finally, we thank Professor Silva for raising these points which we feel have improved the paper considerably.

*Farapti, et al.*
